# Associations between self-reported psychological symptom severity and gut microbiota: further support for the microgenderome

**DOI:** 10.1186/s12888-022-03947-7

**Published:** 2022-04-30

**Authors:** Michael Ganci, Emra Suleyman, Henry Butt, Michelle Ball

**Affiliations:** 1 Psychology Department, Institute for Health and Sport, Victoria University, PO Box 14428, Melbourne, VIC 8001 Australia; 2 Bioscreen Yarraville (Aust) Pty Ltd, Melbourne, VIC Australia

**Keywords:** Gut microbiota, Psychological symptoms, Microgenderome, Brain-gut-microbiota axis, Psychology

## Abstract

**Background:**

Research into the brain-gut-microbiota axis (BGMA) continues to reveal associations between gut microbiota (GM) and psychological symptom expression, inspiring new ways of conceptualising psychological disorders. However, before GM modulation can be touted as a possible auxiliary treatment option, more research is needed as inconsistencies in previous findings regarding these associations are prevalent. Additionally, the concept of the microgenderome, which proposes that GM may interact with sex hormones, has received limited attention in studies using human samples to date. However, such research has demonstrated sex specific associations between GM and psychological symptom expression.

**Method:**

This cross-sectional retrospective study explores associations between GM species (identified through faecal microbial analysis) and symptom severity across four psychological domains (Depressive, Neurocognitive, Stress and Anxiety, and Sleep and Fatigue) for males (*N* = 1143) and females (*N* = 3467) separately.

**Results:**

GM species from several genera including *Bifidobacterium*, *Clostridium*, *Enterococcus*, and *Leuconostoc* were found to be differentially associated with psychological symptom severity for males and females. As such, the findings of the current study provide support for the concept of the microgenderome.

**Conclusion:**

While further research is needed before their implementation in psychological treatment plans, the current findings suggest that modulation of GM at the species level may hold promise as auxiliary diagnostic or treatment options. These findings may give further insight into a client’s presenting problem from a more holistic, multidisciplinary perspective. The clear sex divergence in associations between GM and symptoms give insight into sex discrepancies in susceptibility to psychological disorders.

**Supplementary Information:**

The online version contains supplementary material available at 10.1186/s12888-022-03947-7.

## Background

The proliferation of research into the brain-gut-microbiota axis (BGMA) has continued to demonstrate associations between gut microbiota (GM) and psychological symptom expression [1–8]. It is well established that the trillions of microorganisms in the gut that comprise the GM have co-evolved to share a symbiotic relationship with their human host [9, 10]. The GM is a diverse and highly complex ecosystem comprising of bacteria, fungi, protozoa, viruses, and archaea [11]. GM are believed to influence host health (and alternatively symptom expression) though neuronal, immune, endocrine, and metabolic pathways [12–14]. However, the precise combination of microorganisms that constitutes a ‘healthy’ GM has not been established due to immense intra- and inter-person variability. While more than 2000 microbial species have been identified [10], the number of species within an individual’s GM has been suggested to range between approximately 100–500 [15, 16]. As such, the possible combinations that may exist within and between individuals are exponential.

There is an abundance of research demonstrating the role of GM among psychosocial behaviours including mood, cognition, stress, and sleep in preclinical murine models, albeit studies involving human participants are to date limited [17]. Research involving human participants provides mounting evidence that changes in GM composition, or the abundance of specific microbes, may be associated with psychological symptom expression and disorders. While recent systematic reviews provide evidence of such associations, they also highlight heterogenous and at times contradictory findings, with few taxa being associated with symptom expression across multiple studies [18–22]. One reason for this may be that the majority of studies investigating associations between GM and psychological symptoms investigate the GM at either the genus level or higher. While this provides valuable information, the nuances of more specific species level information is missed. Gaining a more precise understanding of associations between GM and symptom expression at a species level is imperative because the heterogeneity of species within a single genus makes it difficult to prescribe health benefits or detriments at the genus taxonomic rank. Another possible reason for these discrepancies in findings is that there are a number of different techniques commonly used to analyse the presence and abundance of GM [23, 24]. These include culture-based methods, which involve growing selected bacteria and estimating the number of viable (live) cells in a sample, versus DNA based techniques such as 16 rRNA sequencing, and shotgun sequencing which include both live and dead cells. The strengths and weaknesses of these different techniques are discussed elsewhere [25].

### Microgenderome: sex differences in microbe-host relationships

Research supports a complex multidirectional relationship between sex hormones, GM, and immunity which all exist within the broader BGMA communication network [26–28]. Specifically, growing evidence supports the concept of the microgenderome which implies that sex hormones play a role in modulating GM, therefore resulting in sex-specific host-microbiota interactions [29–31]. While sex differences in microbiota composition have mostly been demonstrated in pre-clinical studies, human studies have provided support for the microgenderome [31–38]. Compositional sex differences have been noted in various genera such as *Bacteroides*, *Bilophila*, *Escherichia, and Veillonella* [34, 37]. Meanwhile, Wallis et al. [31] found GM composition to be similar between the sexes, however, *Clostridium*, *Lactobacillus*, and *Streptococcus*, were associated with physical and psychological symptoms in a sex divergent manner. Associations between psychological symptoms and some species of fungi have also demonstrated sex divergence. For example, *Candida albicans* has been found to be associated with cognitive deficits in schizophrenia and bipolar disorder in a sex specific manner [38]. These sex-specific interactions may offer some insight into differing prevalence rates between males and females for various disorders which have been associated with certain gut microbiota profiles such as autism, IBS, anxiety, and depression [39].

### The current study

The current study extends upon work conducted by Wallis et al. [31] who investigated sex specific relationships between microbial genera and symptom expression in a sample of myalgic encephalomyelitis/chronic fatigue syndrome (ME/CFS) sufferers. While Wallis et al. [31] specifically investigated bacterial genera, they suggest the need to investigate the host relationship with microbes at the species level. The current study explored the relationship between an array of microbial species (including bacteria and fungi) in a large, clinically diverse sample.

The aim of the current study was to investigate the relationship between GM at the species level and symptom severity across four psychological symptom domains (Depressive, Neurocognitive, Stress and Anxiety, and Sleep and Fatigue) between males and females. It was hypothesised that the pattern of relationships between GM species and psychological symptom expression would differ between males and females.

## Methods

### Participants

The current study consisted of a retrospectively collected sample of 4610 (1143 males and 3467 females) clinically diverse adult patients ranging in age from 18 to 87 years (*M* = 43.09, *SD* = 13.48). While females clearly outnumbered males, there were sufficiently robust numbers in each sample. Patients were referred to Bioscreen, a Melbourne based laboratory specialising in faecal microbial analysis (FMA), between February 2013 and June 2015. Stool samples were submitted for analysis as part of the investigatory process for intestinal dysbiosis however, the specific diagnostic status of patients were not identified. As such, this is a cross-sectional study of a broad range of potentially clinical and non-clinical presentations. Ethics approval for the current study was obtained from the Victoria University Human Research Ethics Committee (HRE16–071). The Bioscreen Patient Questionnaire included a statement that patients’ data would be used for research purposes, however, patients had the option to ‘opt out’ (by ticking a box), in which case, consent was not provided, and their data was not included in any analyses.

### Sample collection and microbial identification

Stool samples were collected by patients according to directions provided in a Bioscreen FMA kit which was posted to their home. The FMA kit contained a faecal collection tub (anerobic pouch system) with a perforated lid to aid anaerobiasis, which was achieved by activating an Anaero Gen Compact (Oxid, Thermo Fisher, Australia), a zip lock bag, and three icepacks. Samples were transported to the laboratory in polystyrene boxes with three frozen icepacks to maintain a temperature below 12 °C. Stool samples were transported via Express post meaning that sample would arrive at Bioscreen for analysis within 48 h of collection. Faecal samples that were incorrectly collected or transported, or those which were subjected to inaccurate anaerobiosis or refrigeration procedures, were rejected according to Bioscreen’s laboratory protocol.

#### Sample analysis

##### Faecal microbial analysis (FMA)

The FMA process described here was identical to that used in Coulson et al. [40] and Wallis et al. [31] Given that the current study extends on work previously conducted within the same research team at Victoria University by Wallis et al. [31], the procedures outlined below are exactly the same. Given the specificity of FMA, the following information has been sourced from Coulson et al. [40] and Wallis et al. [31]

Once removed from the aerobic collection tub, samples were processed within 10 to 15 min. Between 0.5 and 1.0 g of stool was transferred to 10 mL of 1% glucose-saline buffer. Dilution factor was determined by the difference in the weight of the glucose-saline buffer with and without the sample. One hundred and one thousand-fold dilutions (beginning from 10^− 1^ to 10^− 7^ of homogenised faecal samples were prepared. Dilutions (10 and/or 1 μL amounts) were transferred onto previously dried Columbia horse blood agar (Oxid), chromogenic medium (Oxid), colistin and nalidixic acid blood selective agar (Oxid), and chloramphenicol-gentamicin selective Sabouraud agar for aerobic incubation. Aerobic media were incubated at 35 °C for 48 h. Columbia horse blood haemin agar and Raka Ray medium were used for anaerobic incubation in anaerobic jars (Oxid) for a duration of 4 days. A stereomicroscope was used to examine aerobic and anaerobic culture plates for a minimum of 20 min per plate before bacterial identification. Each colony from each medium was microscopically examined and the colony/viable count were quantified. To assess purity prior to identification, similar morphotypes were sub-cultured onto horse blood agar.

Following these purity checks (overnight), index bacterial colonies were transferred to a target polished steel plate (MSP 96, Bruker Daltonics Inc.) for drying under exhaust ventilation in a Class II Biohazard Hood (Gelman Sciences Australia) at room temperature. Air-dried samples were then subject to protein extraction with 1 μL 70% formic acid (Sigma). After again being allowed to dry under exhaust ventilation, samples were overlain with 1 μL of matrix solution (saturated solution of α-cyano-4-hydroxycinnamic acid [HCAA] in a mixture of 47.5% ultra-pure water, 2.5% trifluoracetic acid, and 50% acetonitrile). Once dried, samples were then analysed using a Microflex matrix assisted laser desorption ionization-time of flight (MALDI-TOF) mass spectrometer (Bruker Daltonik GmbG, Leipzig, Germany) equipped with a 60 Hz nitrogen laser. Spectra were recorded in the positive linear mode for the mass range of 2000 to 20,000 Da at maximum laser frequency. Raw spectra were analysed using the default settings of the MALDI Biotyper 3.0 software package (Daltonik GmbH, Bremen, Germany) which can detect approximately 5000 species. The most prevalent microorganisms were quantified as colony forming units (CFU)/g.

##### Bioscreen patient questionnaire (BPQ)

The BPQ is an 88-item questionnaire developed by Bioscreen administered to patients as part of Bioscreen’s standard procedure. Items on the BPQ are similar in nature to other symptom checklists which relate to diverse symptomatology and patients are asked to report the frequency (over the past 12 months) and severity (over the past 7 days) of their symptoms on a five-point Likert scale ranging from zero to four, with higher scores indicating higher ratings of frequency/severity. For the purposes of the current study, only symptom severity over the past 7 days was assessed, given the underlying assumption of a temporal relationship between the presence of specific GM and symptom expression. The BPQ has been used in previous studies such as Wallis et al. [31] who used clinically derived factors, and Ganci et al. [41] who subjected the severity items of the BPQ to an exploratory factor analysis. While 10 factors were derived, only the four psychological symptom domains were included in the current study which include Depressive symptoms (6 items; Cronbach’s α = 0.894), Neurocognitive symptoms (8 items; Cronbach’s α = 0.938), Stress and Anxiety symptoms (9 items; Cronbach’s α = 0.874), and Sleep and Fatigue symptoms (6 items; Cronbach’s α = 0.853). Possible score ranges for each symptom domain can be found in Table 1, with higher scores indicating the endorsement of greater symptom severity.

**Table 1:** Descriptive information for males and females relating to differences in symptom severity

Symptom domain (possible range)	Males			Females		
	*n*	*M*	*SD*	*n*	*M*	*SD*
Depressive (0–24)	1071	7.469	6.266	3265	9.055	6.702
Neurocognitive (0–32)	1074	10.077	8.705	3291	12.336	8.922
Stress and Anxiety (0–36)	1059	8.062	7.558	3217	10.094	8.144
Sleep and Fatigue (0–24)	1085	10.129	6.440	3297	12.351	6.320

##### Data handling and statistical design

Both aerobic and anaerobic bacterial species and fungi were analysed in the current study. Data regarding intestinal protozoa were investigated in a previous study [41] and are not included here. Viruses, which are also part of the gut microbiota, were not analysed as they were not included in the retrospectively collected data.

A total bacterial count CFU/g was calculated by adding the viable CFU/g counts across all bacterial species, with total aerobe CFU/g and total anaerobe CFU/g counts also being calculated by adding only aerobic or anaerobic bacteria respectively. The total fungal CFU/g count was calculated in the same way, by adding the CFU/g counts for each fungal species.

**Fig. 1:**
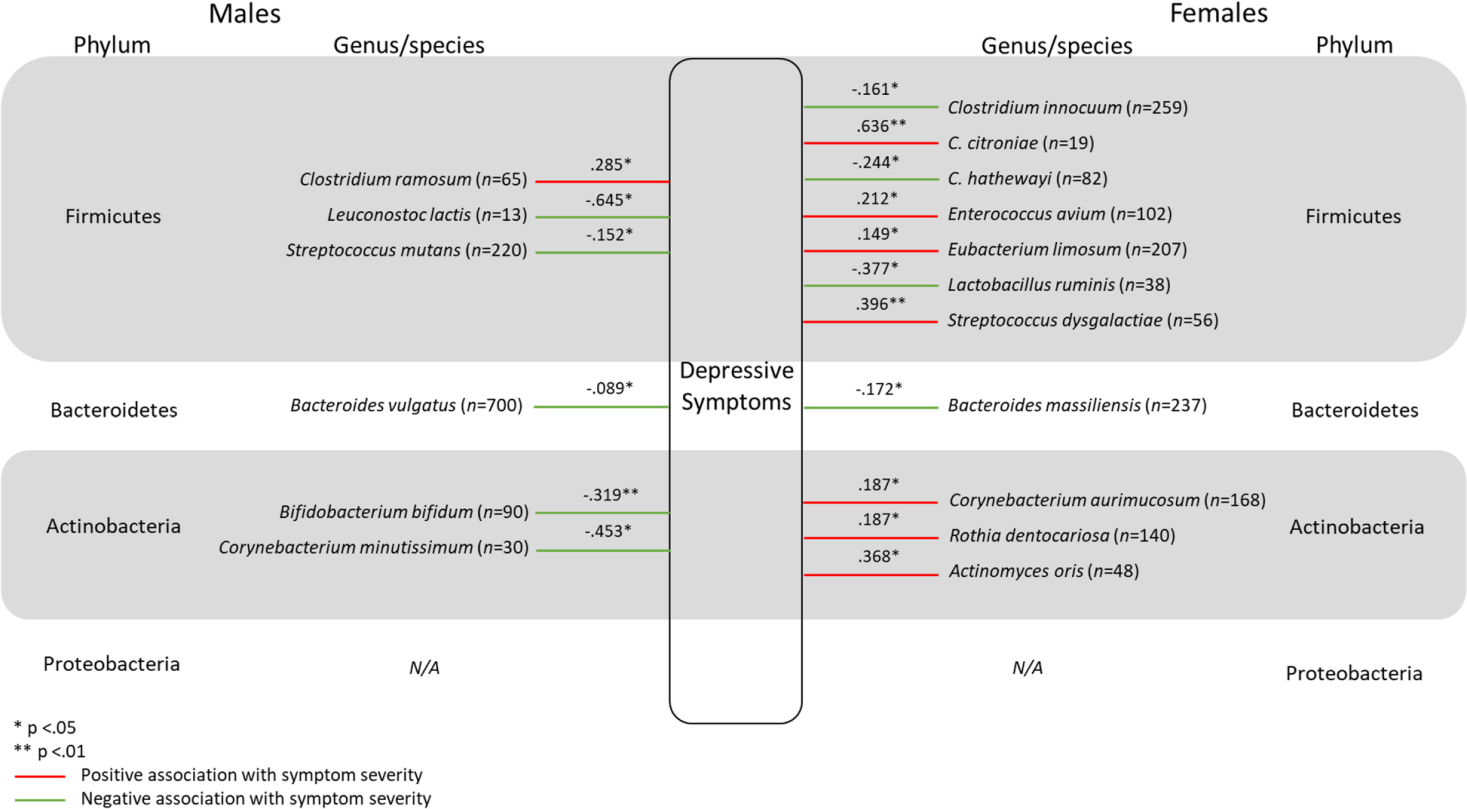
Statistically significant associations between gut microbes and self-reported Depressive symptom severity for males and females. All values reported in Figure 1 are r values which have been converted from τ_b_

**Fig. 2:**
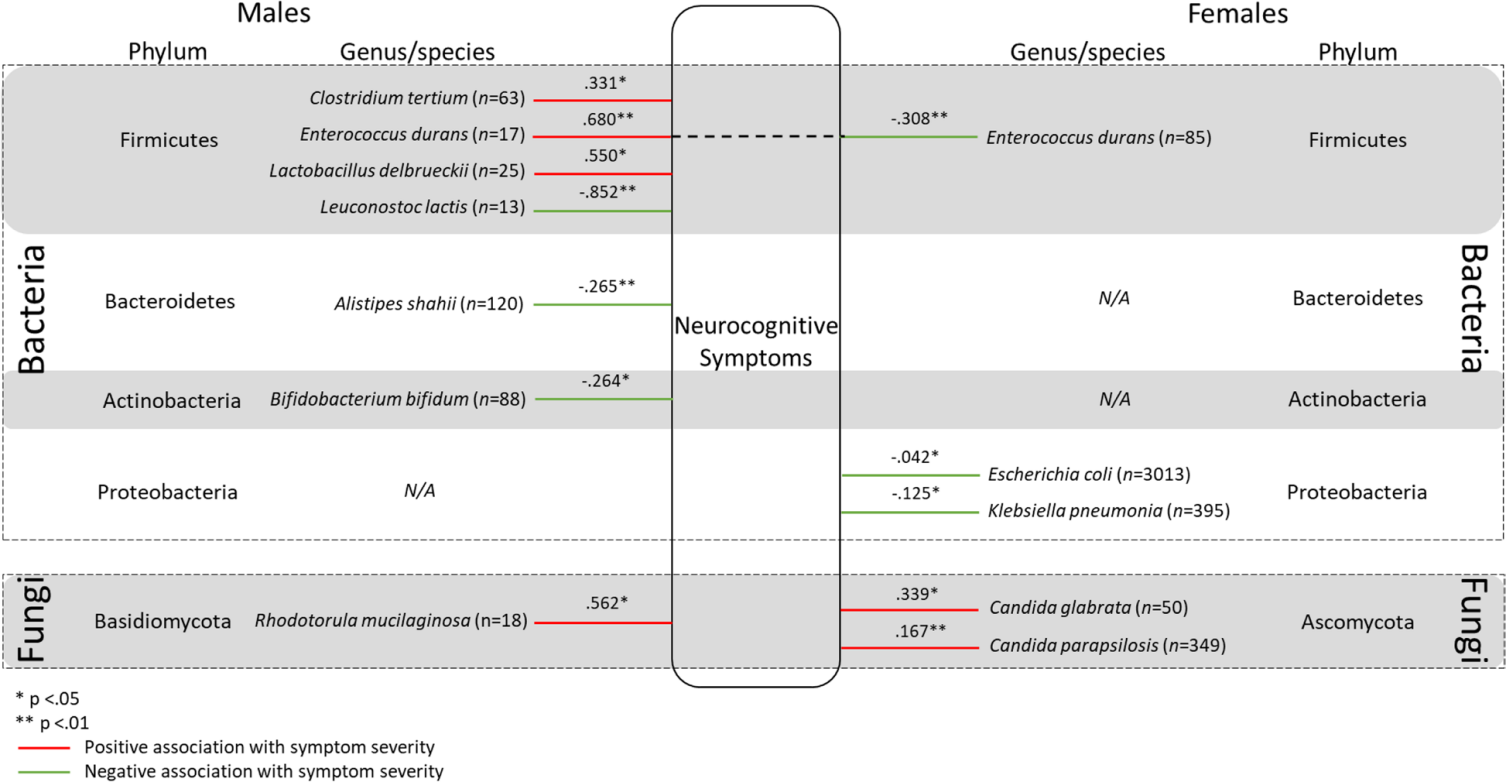
Statistically significant associations between gut bacteria and self-reported Neurocognitive symptom severity for males and females. All values reported in Figure 2 are r values which have been converted from τ_b_

**Fig. 3:**
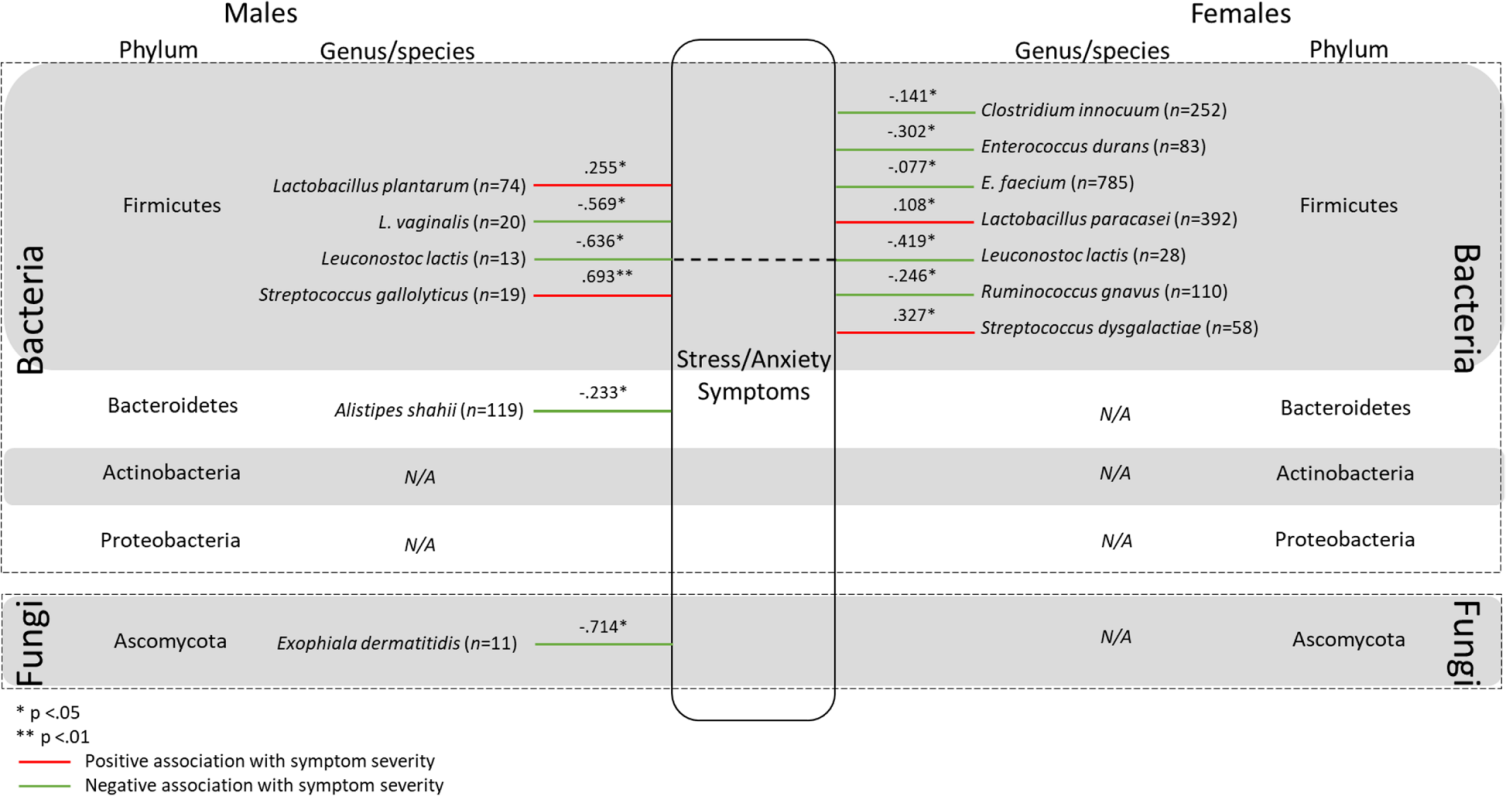
Statistically significant associations between gut bacteria and self-reported Stress and Anxiety symptom severity for males and females. All values reported in Figure 3 are r values which have been converted from τ_b_

Four hundred and ninety-five bacterial and fungal species were identified in the overall sample of Bioscreen patients over the two and a half years of testing between 2013 and 2015. Of the 495 bacterial and fungal species identified, 132 species were found to have a viable count in only a single patient, and a further 188 species were identified in between only two and fourteen patients (< 0.5% of the patient sample). Therefore, a total of 175 species (species that had a viable count in ≥15 patients) were analysed to determine if there were significant differences between the viable CFU/g count in males and females. Pairwise deletion was used to deal with missing data. Given that many variables had a large proportion of missing data (ranging from 32 to 99.98% missing), data imputation methods would not have been appropriate [42]. Furthermore, Kaul et al. [43] refers to missing data in microbiome research as “structural zeros” which are due to an individual’s underlying biology. For example, if an individual harbours a combination of between 100 and 500 species of GM [15, 16], but over 2000 species have been identified [10], it is highly unlikely that any two individuals would have the same combination of microorganisms, resulting in high levels of missing data as observed in the current study. Therefore, Kaul et al. [43] argue that the use of typical imputation methods would be erroneous. As such, the sample size for comparisons of CFU/g between males and females, and associations between microbial species and psychological symptom severity varied by species. While many of the microbial species analysed were detected in a substantive number of patients, several other microbes were detected in very few patients (see Table 2 and Figs. 1, 2, 3, & 4). The retrospective nature of the current study precluded the collection of further data. For example, age could not be considered due to missingness in the data which would mean that sample sizes would be even further reduced. Moreover, the retrospective dataset did not include information regarding an individual’s life stage (i.e. menopause).

**Table 2:** Statistically significant results of Mann-Whitney U test assessing difference in the viable CFU/g count of species between males and females

Species	Male		Female		*U*	*z*	*p*	*r*
	*n*	Median (*Md*)	*n*	Median (*Md*)				
*Alistipes finegoldii*	70	7.215*10^9^	274	3.835*10^9^	7489	−2.829	.005	.153
*Bacteroides uniformis*	567	7.400*10^9^	1841	6.090*10^9^	481,924	−2.763	.006	.056
*Bacteroides vulgatus*	745	8.000*10^9^	2287	6.070*10^9^	775,833.5	−3.666	<.001	.067
*Bifidobacterium animalis*	169	1.350*10^8^	665	9.430*10^7^	48,876.5	−2.616	.009	.091
*Collinsella aerofaciens*	631	7.020*10^9^	1710	5.315*10^9^	486,257	−3.669	<.001	.076
*Candida albicans*	314	2250	914	1380	130,007	−2.489	.013	.071
*Lactobacillus gasseri*	103	2.130*10^6^	272	7.245*10^5^	11,220.5	−2.975	.003	.154
*Lactobacillius rhamnosus*	83	2.070*10^6^	311	1.050*10^6^	11,019.5	−2.047	.041	.103
*Lactobacillus salivarius*	37	7.490*10^6^	134	1.860*10^6^	1932	−2.052	.040	.157
*Parabacteroides goldsteinii*	13	4.800*10^8^	58	1.860*10^9^	237	−2.082	.037	.247
*Ruminococcus gnavus*	30	3.655*10^9^	124	1.845*10^9^	1397	−2.112	.035	.170
*Streptococcus salivarius*	533	5.870*10^6^	1337	3.000*10^6^	305,494	−4.821	<.001	.111
*Streptococcus parasanguinis*	523	3.060*10^6^	1404	1.980*10^6^	322,444.5	−4.116	<.001	.094
*Streptococcus gordonii*	64	2.640*10^6^	139	1.010*10^6^	3320	−2.901	.004	.204

**Fig. 4:**
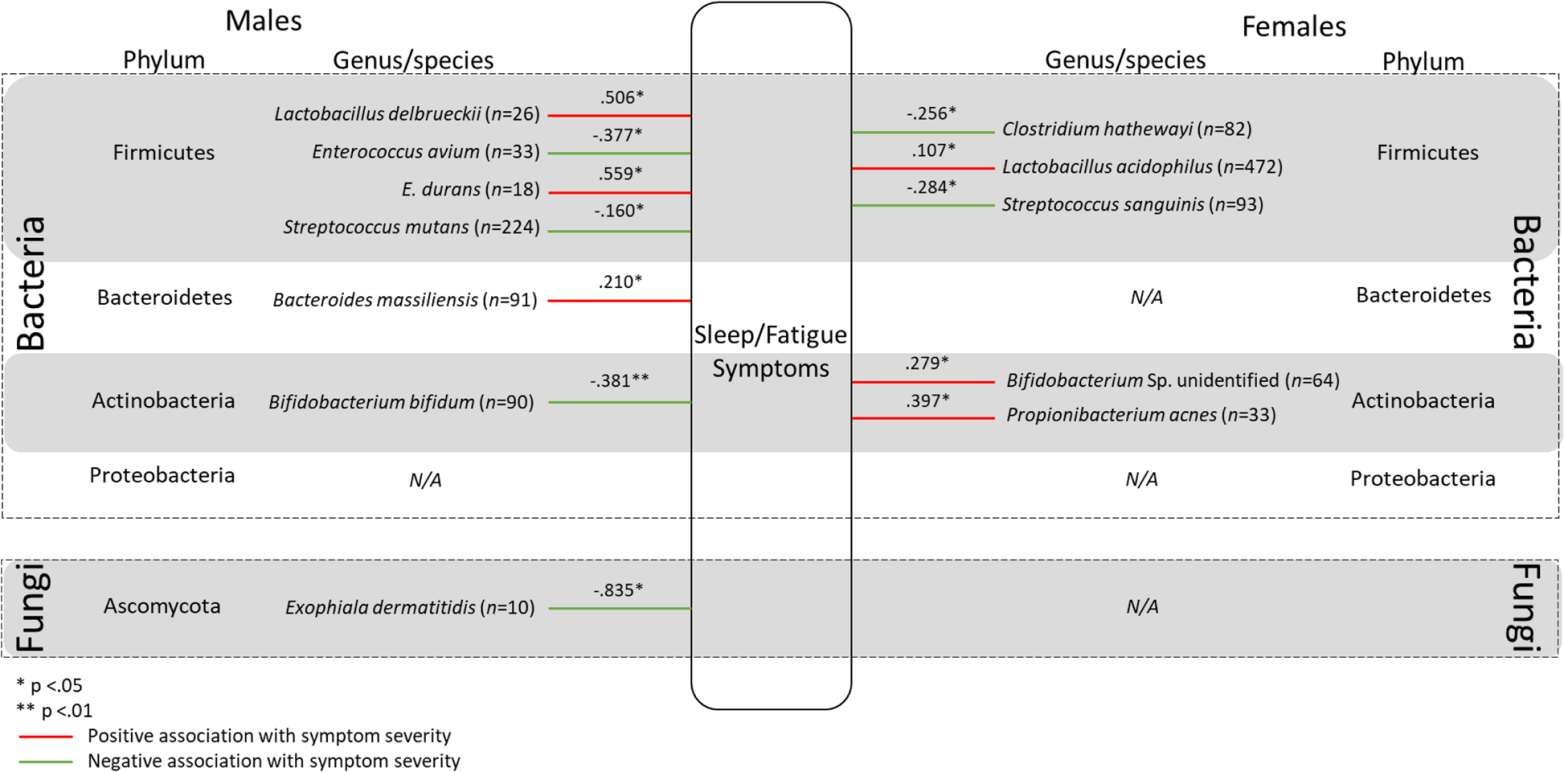
Statistically significant associations between gut bacteria and self-reported Sleep and Fatigue symptom severity for males and females. All values reported in Fig. 4 are r values which have been converted from τ_**b**_

As the assumptions of parametric statistics were violated for CFU/g counts, namely normality and the presence of outliers, sex differences in microbial counts were analysed using a series of Mann-Whitney *U* tests. While nonparametric tests have less statistical power, it is argued that when the assumption of normality is violated or when outliers are present, nonparametric tests are clearly the correct choice [44, 45], especially in small samples [46, 47]. While the overall sample size of the current study was large, sub-samples of individuals with specific microorganisms were often small within each sex. While there is no consensus over what is considered to be a minimum sample size, particularly for nonparametric tests, it has been suggested that a Mann-Whitney *U* test requires a minimum of 4 participants per group (*N* = 8) before having the possibility of rejecting the null hypothesis [48]. However, given that there is consensus that smaller samples provide less valid findings [49–51], a higher minimum number of cases (*n* ≥ 10) was used as the benchmark for consideration in the current study. The authors acknowledge the limitations of the current data set and suggest that the results of the current study be interpreted with caution, and should be considered as exploratory. As such, no adjustments were made to the alpha levels. While the issue of alpha value adjustment is somewhat contentious, Rubin [52] argues that it is not necessary for exploratory analyses. It is also argued that alpha adjustments are only necessary when multiple tests are used to test the same hypothesis within the exact same population [52], which was not the case in the current study.

Reported *z* scores from the Mann-Whitney *U* analyses were used to calculate an approximate value of *r* as a measure of effect size using the formula (1):

1 $$r=\frac{z}{\sqrt{N}}$$

Further analyses using a series of Kendall’s Tau-b (τ_**b**_) correlations were performed to determine the monotonic relationship between gut microbiota and psychological symptom severity across the domains of depressive, neurocognitive, stress and anxiety, and sleep and fatigue symptoms. Some authors have suggested that Kendall’s τ_**b**_ may draw more accurate generalisations compared to Spearmans rho [53]. Correlations were interpreted only in cases where the sample size was at least 10 in both males and females (given that correlations between GM and psychological symptom severity were run separately for each sex) as it has been suggested that Kendall’s τ_**b**_ performs reasonably well with small samples (*n* ≥ 10), but also under a variety of other conditions and sample sizes [54]. As such, 152 GM species were analysed to determine correlations between GM and psychological symptom severity. Long and Cliff [54] go so far as to suggest that Kendall’s τ_**b**_ should be considered a worthy measure of correlation in its own right, and not only as an alternative to Pearson’s correlation coefficient, also making it a suitable choice for larger samples.

In order to aid in the interpretation of the strength of the relationships identified using Kendall’s τ_**b**_, τ_**b**_ values were converted to *r* using formula (2) [55].

2 $$r=\mathit{\sin}\left(.5\ \pi\ \tau \right)$$

All values presented in Figures throughout the results section are *r* values which were converted from Kendall’s τ_**b**_ values using (2). See Table S1 for the original Kendall’s τ_**b**_ values, along with the associated *r*, *r*^*2*^, *n*, Fisher’s z transformation (*z*_*r*_), and *p* values for each species associated with the four psychological symptom factors for males and females.

In cases where the same species of gut microorganism was found to be associated with a psychological symptom factor in both males and females, the strength of the relationship between that microbe and symptom severity for each sex was compared using Fisher’s *z*-transformation (Z_r_) of *r* using formula (3).

3 $${z}_r= \frac{1}{2}\ {ln \left[ (1+r)/(1-r) \right]}$$

Once the *r* for males and females were converted into *z* scores, formula (4) was used to determine if there was a significant difference in the strength of the associations.

4 $${\mathrm{z}}_{\mathrm{obs}}=\frac{z_{1^{-}}{z}_2}{\sqrt{\frac{1}{N_1-3}+\frac{1}{N_2-3}}}$$

## Results

### Sex differences in self-reported symptom severity

Presented in Table 1, are the mean and standard deviation symptom severity scores for the four psychological domains measured. As can be seen, females self-reported greater symptom severity across all four psychological symptom domains. A series of independent samples *t*-tests were run to determine whether these differences were statistically significant.

It was found that females’ self-reported symptom severity was significantly higher compared to males for Depressive symptoms, *t* (1934.423) = −7.062, *p* < .001, *d* = .244, Neurocognitive symptoms, *t* (4363) = − 7.247, *p* < .001, *d* = .256, Stress and Anxiety symptoms, *t* (1928.529) = − 7.440, *p* < .001, *d* = .259, and Sleep and Fatigue symptoms, *t* (4380) = −9.996, *p* < .001, *d* = .348. According to suggested benchmarks [56], the effect size for each symptom domain was small, suggesting that sex alone only accounts for a small proportion of variance in symptom severity.

### Sex differences in bacterial composition

Mann-Whitney *U* tests showed that at the most global level, statistically significant sex differences were found between males (*n* = 1143) and females (*n* = 3467) in regards to viable total bacterial count (*Mdn*_males_ = 2.98 × 10^10^, *Mdn*_females_ = 2.64 × 10^10^, *U* = 1,853,660, *z* = − 3.259, *p* = .001, *r* = .048), total aerobe count (*Mdn*_males_ = 5.77 × 10^7^, *Mdn*_females_ = 4.28 × 10^7^, U = 1,848,775.5, *z* = − 3.398, *p* = .001, *r* = .050), and total anaerobe count (*Mdn*_males_ = 2.96 × 10^10^, *Mdn*_females_ = 2.60 × 10^10^, *U* = 1,853,741, *z* = − 3.257, *p* = .001, *r* = .048), with males demonstrating higher median CFU/g counts in all three instances. Additionally, the total fungal CFU/g count was also found to be significantly higher in males (*n* = 1074) compared to females (*n* = 3281; *Mdn*_males_ = 186.500, *Mdn*_females_ = 110, U = 1,687,589.500, *z* = − 2.189, *p* = .029, *r* = .033). Using McGrath and Meyer’s [57] guidelines for the interpretation of *r* (based on Cohen [56]), the effect sizes of these differences were all very small, suggesting that the statistical significance was likely a statistical artifact due to the large sample.

Of the 175 species analysed using Mann-Whitney *U* tests, the viable CFU/g count of 14 species (13 bacterial and one fungal) were found to be significantly different in abundance between males and females. The species identified as exhibiting sex differences in viable CFU/g counts are displayed in Table 2.

As can be seen in Table 2, for microbes which showed a significant difference in abundance between males and females, males had a higher count in all but one GM species, *Parabacteroides goldsteinii*.

While statistically significant, the effect sizes of the differences in CFU/g between males and females were low or negligible, as can be seen in Table 2. This suggests the statistical significance is either due to statistical artifact, or that sex alone only accounts for a very small portion of variance in microbial CFU/g counts between males and females. Considering that only 14 out of 175 species of gut microbes analysed demonstrated a significantly different abundance between males and females, and the low to negligible effect sizes, the overall snapshot provided by these results suggests a predominantly similar microbial composition between the sexes.

### Sex dependent relationships between gut microbes and psychological symptomatology

While composition was found to be mostly similar between males and females, the results showed that associations between gut microbes and psychological symptom severity varied in a sex dependent manner across all four psychological symptom factors. Of the 152 GM species analysed, 39 different microorganisms (35 bacterial and four fungal) were found to be associated with at least one of the four psychological symptom domains.

Positive associations between a microbial species and psychological symptoms indicate that an increase in the rank of viable CFU/g count was monotonically associated with an increase in the rank of symptom severity. That is, a positive correlation indicates that the species is associated with more severe symptoms. Conversely, a negative association indicates that an increase in the rank of viable CFU/g count was monotonically associated with a decrease in the rank of symptom severity. That is, a negative correlation indicates that the species is associated with less severe symptoms.

### Depressive symptoms

Relationships between GM and Depressive symptom severity differed in a sex dependent manner. There were more bacterial species associated with Depressive symptom severity in females compared to males, particularly from within the Firmicutes phylum. *Clostridium ramosum* was the only microbe found to be positively associated with Depressive symptom severity in males and *Leuconostoc lactis* demonstrated the strongest negative relationship with symptom severity. For females, *C. citroniae* showed the strongest positive association with symptom severity, while *Lactobacillus ruminis* demonstrated the strongest negative relationship with symptom severity. See Fig. 1 for all statistically significant associations between GM and Depressive symptom severity in males and females. Statistically non-significant correlations are not included in the figure.

### Neurocognitive symptoms

The associations between GM and Neurocognitive symptom severity again showed a different pattern between males and females. *L. lactis* once again showed the strongest negative association with symptom severity in males. On the other hand, *Enteroccocus durans* demonstrated the strongest positive association with symptom severity in males. Conversely, in females, *E. durans* demonstrated a weak negative association with symptom severity. Not only was the direction of the association contrasting between males and females for this species, but there was also a significant difference in the strength of the association between sexes (z_obs_ = 7.479, exceeding the critical value of 1.96). See Fig. 2 for all statistically significant associations between gut bacteria and neurocognitive symptoms in males and females. Statistically non-significant correlations are not included in the figure.

### Stress and anxiety symptoms

The overall pattern of associations between GM and Stress and Anxiety symptom severity was largely distinct between males and females. *L. lactis* again showed a strong negative association with symptom severity in males. Demonstrating sex consistency, *L. lactis* also showed a moderate negative association with symptom severity in females. The difference in the strength of the associations between males and females for *L. lactis* did not exceed the critical value of 1.96 and was therefore not statistically different (*z*_*obs*_ = − 1.634). See Fig. 3 for all statistically significant associations between GM and Stress and Anxiety symptom severity in males and females. Statistically non-significant correlations are not included in the figure.

### Sleep and fatigue

Again, the pattern of associations between GM and Sleep and Fatigue symptom severity was different between males and females. Several GM demonstrated similar associations with Sleep and Fatigue symptom severity as they did with other symptom domains including negative associations with *B. bifidum* and *Exophiala dermatitidis* and positive associations with *L. delbrueckii* and *E. durans*. See Fig. 4 for statistically significant associations between gut microbes and sleep and fatigue symptom severity for males and females. Statistically non-significant correlations are not included in the figure.

## Discussion

The aim of the current study was to investigate the relationship between GM and symptom severity across four psychological domains (Depressive, Neurocognitive, Stress and Anxiety, and Sleep and Fatigue symptoms) and to assess whether these relationships differed between males and females. The hypothesis that the pattern of relationships between GM and psychological symptom severity would vary between males and females was supported. There was clear divergence in the patterns and species of GM associated with symptom severity in males and females across all four psychological symptom domains. As such, these findings provide further support for the concept of the microgenderome in a human sample.

The current study found that females endorsed greater symptom severity across all four psychological domains which is consistent with previous research [58–62]. Barsky et al. [59] suggest that socialised gender expectations may explain differences in symptom reporting between males and females. Others have suggested that sex differences in symptom expression are based on interactions between sex hormones and serotonergic and glutamate systems [63, 64]. Sex differences in immune functioning have also been proposed to explain the increased prevalence of mood and anxiety disorders in females [65, 66]. However, the small effect sizes found in the current study suggest that sex alone explains only a small amount of variance in self-reported psychological symptom severity.

Of the 39 species found to be associated with psychological symptom severity, only *Ruminococcus gnavus* was found to have significantly different counts between males and females (as seen in Table 2). Gao et al. [33] found the genus *Ruminococcus* to vary according to gender, finding it to be more abundant in females, which has also been reflected in murine models [67]. However, the current study found *R. gnavus* to be more abundant in males. Although somewhat contradictory, these findings suggests that Ruminococci species may be particularly vulnerable to the influence of sex hormones. This discrepancy may be due to the fact that the current study investigated sex differences at the species level whereas Gao et al. [33] reported differences at the genus level. This means that perhaps other species within the *Ruminococcus* genus may collectively make the genus more abundant in females. This possibility supports the need for future research to be conducted at the more specific species level to provide a clearer and more precise indication of the associations between GM, sex hormones, and symptom expression.

All other species found to have a relationship with symptom severity in the current study showed a similar abundance in males and females, demonstrating that sex-divergent associations between GM and symptom severity were not due to underlying differences in GM composition. This finding is similar to that of Wallis et al. [31] who also found sex specific correlations between bacterial genera and a variety of physical and psychological symptom domains despite similar bacterial composition between the sexes.

Part of the rationale for the current study was the need to explore associations between GM and psychological symptoms at the species level in contrast to the majority of previous research focusing of genus level and above. The results of the current study show that, for example within the *Clostridium* genus, certain species were positively associated with symptom severity (*C. citroniae, C. ramosum,* and *C. tertium*), while other species within this same genus were negatively associated with symptom severity (*C. innocuum* and *C. hathewayi*). These findings are consistent with previous research having shown similar disparities between *Clostridium* species [68, 69]. If measured at the genus level, these nuances would have been missed, and important associations may have been masked.

### Depressive symptoms

Previous findings have established that individuals with depression have markedly different GM composition [3, 18–22, 32]. Depression is considered to be the leading cause of disability worldwide [70]. As such, research into the BGMA offers valuable information regarding possible biological contributions to the pathogenesis of the disorder [71]. Furthermore, the differing patterns of associations between GM and depressive symptoms in males and females may offer insight into sex differences in prevalence rates of the disorder.

The findings of the current study demonstrated a greater number of species were found to be associated with Depressive symptoms in females compared to males. *Clostridium ramosum* was the only bacterial species found to be positively associated with Depressive symptom severity for males, showing a weak association. On the other hand*, Leuconostoc lactis* was found to have a moderate negative association with Depressive symptom severity in males. *L. lactis* was also found to be negatively associated with both Neurocognitive and Stress and Anxiety symptom severity in males. The seemingly protective nature of *L. lactis*, at least for males, may be explained by previous studies which have found the species to have anti-inflammatory properties [72, 73], to inhibit the effects of pathogenic bacteria and fungi [74], and to be associated with improved cardiovascular health [75]. For females, *Clostridium citroniae* was positively associated with symptom severity, however, *C. innocuum* and *C. hathewayi* were negatively associated. As such, this demonstrates the need for research to be conducted at the species level to provide more detailed and precise information regarding GM and their association with symptom expression. *Lactobacillus ruminis* also demonstrated a negative association with symptom severity in females.

For males, *Bifidobacterium bifidum* was found to be negatively associated with Depressive symptom severity and was also found to be negatively associated with both Neurocognitive and Sleep and Fatigue symptom severity. These findings are in line with previous research purporting health benefits of *B. bifidum* [76–78]. The results are also in line with those of Aizawa et al. [79] who, at the genus level, found *Bifidobacterium* to be reduced in those diagnosed with depression. For females, *B. bifidum* was not found to be associated with any of the four psychological symptom domains measured, suggesting that the species’ health promoting effects may be sex dependent. The current findings support the notion that species from the *Bifidobacterium* genus act in a sex dependent manner [80]. However, the sex effect was reversed in the current study as Luk et al. [80] found that, at the genus level, *Bifidobacterium* ameliorated anxiety like behaviours only in female mice. It is possible that measurement of GM at the broader genus level masks underlying sex differences in specific species within a genus, warranting further research at the species (and/or strain) level.

In females, *Corynebacterium aurimucosum*, *Rothia dentocariosa*, and *Actinomyces oris* (species belonging to the Actinobacteria phylum) demonstrated a positive association with symptom severity. This finding is in keeping with those of Chen et al. [32] who found a greater abundance of phylum Actinobacteria in females diagnosed with depression, but not males. Sex divergent associations were demonstrated within the *Corynebacterium* genus as *C. minutissimum* was found to be negatively associated with Depressive symptoms in males. *Corynebacterium* species have previously been demonstrated to produce serotonin in culture mediums [81] which may generally explain their association with Depressive symptoms, however it does not explain why these effects were sometimes reversed between the sexes. Although further research is necessary to clarify the role of *Corynebacterium* in serotonin production, the findings of the current study demonstrate that the role of sex hormones should be considered in any such investigations.

Demonstrating some consistency between the sexes, *Bacteroides* species (*B. vulagtus* in males and *B. massiliensis* in females) were also found to be negatively associated with Depressive symptom severity. This finding was in line with those of Jiang et al. [3] who found *Bacteroides* at the genus level to be depleted in patients with depression, however this is in contrast to the findings of Liu et al. [82] and Rong et al. [7] These inconsistencies suggest the need for further investigation into association between *Bacteroides* species and Depressive symptoms.

### Neurocognitive symptoms

While neurocognitive symptoms are not themselves a specific disorder, they occur across numerous physiological and psychological disorders [83–85]. Brain fog is a form of neurocognitive impairment which features mental confusion, impaired judgement, deficits in short term memory, and difficulty concentrating [86]. Given the associations reported in the literature between brain fog and bacterial overgrowth [86], the current investigation of potential sex differences in the pattern of associations was largely exploratory. Severance et al. [38] found *Candida albicans* IgG to be differentially associated with neurocognitive symptoms in males and females with schizophrenia and bipolar disorder. The pattern of associations between GM species and Neurocognitive symptom severity in the current study also differed between males and females. For males, *Clostridium tertium*, *Enterococcus durans*, and *Lactobacillus delbrueckii* were positively associated with symptom severity. As aforementioned, *L. lactis* was found to be negatively associated with symptom severity in males. Demonstrating a clear sex difference, while *E. durans* was positively associated with Neurocognitive symptoms severity in males, this species was found to be negatively associated with symptom severity in females. This is a potentially important finding because *E. durans* has been studied for its probiotic potential and has been found to possess anti-inflammatory properties [87], and may also increase the known anti-inflammatory species *Faecalibacterium prausnitzii* [88]. While there is extremely limited research investigating sex differences in the function of *E. durans*, Wallis et al. [31] found sex specific associations between *Enterococcus* at the genus level and ME/CFS symptoms. Interestingly, in contrast to the findings of this paper, Wallis et al. [31] found a positive relationship between symptoms and *Enterococcus* only in females. Taken together, these somewhat contradictory findings suggest that *Enterococcus* species may interact with sex hormones in complex ways which warrant further investigation.

*Alistipes shahii* was found to be negatively associated with the severity of both Neurocognitive, and Stress and Anxiety symptoms, but only in males. At the genus level, *Alistipes* have been associated with psychological disorders such as depression [89, 90] and ASD [91, 92], however findings have been contradictory in terms of whether *Alistipes* are more abundant in symptomatic or healthy groups. Several hypotheses have been proposed regarding how *Alistipes* may influence symptom expression including inflammation, interference with serotonergic signalling, and metabolite production [93]. Parker et al. [94] specify a need for further research into the genus given the contradictory findings regarding its potential protective or pathogenic role. The current findings suggest that further investigations would benefit from being conducted at the species level which would provide more detailed and precise information about the protective and pathogenic potential of different members of the *Alistipes* genus. Further, the results of the current study suggest that sex differences should be considered in future studies as sex hormones may provide further insight and clarity into the role of *Alistipes* in psychological health and disease.

Demonstrating sex consistency, fungi were found to be positively associated with Neurocognitive symptom severity in both males (*Rhodotorula mucilaginosa*) and females (*Candida glabrata* and *C. parapsilosis*). This finding is consistent with previous research which has demonstrated an increased abundance of *Candida* in individuals diagnosed with neurocognitive disorders [92, 95]. It is proposed that *Rhodotorula* species in the gut are likely to provide benefits to the host through the production of various nutrients, and potentially neutralising the toxins of pathogens [96]. However, Hof [96] also proposes that *Rhodotorula* species could be detrimental in large numbers as they are able to metabolise short-chain fatty acids, thereby reducing their availability. *Rhodotorula* may also have a detrimental effect on certain pathophysiological groups (such as critically ill patients) or immunocompromised hosts. *Candida* species, including *C. glabrata* and *C. parapsilosis*, are commonly found in the human gastrointestinal and genitourinary tracts and skin [97], however, they appear to be opportunistic pathogens, having detrimental effects on immunocompromised individuals [98]. Specifically, *C. parapsilosis* has been found in greater abundance in patients with Rett syndrome and is believed to be involved in chronic inflammation [95]. As such, the results of the current study are consistent with previous research demonstrating a potential association between these *Candida* species and Neurocognitive symptom expression.

### Stress and anxiety symptoms

Both stress and anxiety disorders are prevalent as clinical entities with a high disease burden, and also occur at subclinical levels in the population with considerable impairment in functioning [99, 100]. They too have been associated with changes in microbial composition of the gut [4, 6]. Results of the current study show that for females, only species belonging to the Firmicutes phylum were found to be associated with Stress and Anxiety symptom severity. *Clostridium innocuum*, *E. durans*, *E. faecium*, *L. lactis*, and *Ruminococcus gnavus* were found to be negatively associated with Stress and Anxiety symptom severity. The finding regarding *R. gnavus* is inconsistent with Jiang et al. [4] who found an increased abundance of the species in those with GAD, however they did not investigate sex differences. In males, *L. lactis* was also found to be negatively associated with Stress and Anxiety symptom severity, demonstrating some sex consistency. *Lactobacillus vaginalis*, *Alistipes shahii*, and *Exophiala dermatitidis* were also negatively associated with Stress and Anxiety symptom severity in males.

*E. dermatitidis* is a black yeast that has been described as an opportunistic pathogen [101] which is typically found in domestic settings on plastics and rubbers in humid environments such as saunas and dishwashers [102]. Lavrin et al. [102] discussed the possible role of *E. dermatitidis* in neurocognitive disease such as Alzheimer’s, however, their study focused on a particular strain of *E. dermatitidis* (EXF – 10,123). *E. dermatitidis* has also been associated with systemic infection [103] and cirrhosis of the liver [104]. These findings are related to circumstances where *E. dermititidis* is found outside of the gut, in some instances crossing the blood-brain-barrier into the brain. An explanation for the contrasting results between the current study and that of Lavrin et al. [102] and others who have found the microorganism to be associated with severe negative health outcomes may be that *E. dermatitidis* exerts a different influence via the BGMA as a gut microbe, as opposed to direct contact with the CNS. Given the stark contrast between the current findings and previous research regarding *E. dermatitidis*, together with the common presence of *E. detmatitidis* in household appliances such as dishwashers [105], these finding calls for further investigation.

*L. plantarum* and *Streptococcus gallolyticus* were positively associated with symptom severity in males, while *L. paracasei* and *S. dysgalactiae* were found to be positively associated with symptom severity in females. The finding that *Lactobacillus* species were positively related to Stress and Anxiety symptom severity is consistent with the findings of Jiang et al. [4] and somewhat consistent with Taylor et al. [6] who only found *Lactobacillus* to be positively associated with anxiety symptoms in females. *Lactobacillus* species are generally considered to be probiotic, with *L. plantarum* and *L. paracasei* both included in popular over the counter probiotic supplements. These findings are similar to several others who have demonstrated a positive association between *Lactobacillus* and the expression of various psychosocial symptoms [4, 6, 31, 86, 92, 106]. Most species belonging to the *Lactobacillus* and *Streptococcus* genera produce D-lactic acid, which may be associated with altered mental states [31, 86, 107]. Taken together, these findings bring into question whether such probiotic supplements should be used indiscriminately, let alone publicised as health promoting. Given that the current study is associative only, and information regarding probiotic use and health status was unavailable, it may be that those experiencing high levels of anxiety were taking a probiotic supplement, hence the increased abundance of these species. As such, it is not the contention of this study to suggest that *Lactobacillus* species are pathogenic, however, it does demonstrate that further research is called for.

### Sleep and fatigue symptoms

Sleep plays an important role in maintaining health [108]. Many physiological and psychological disorders are associated with poor sleep [109, 110]. While having received less attention in the literature thus far, evidence shows an association between GM and sleep physiology [1, 111]. The current study adds to this literature by demonstrating sex divergent relationships between GM and severity of Sleep and Fatigue symptoms. *Lactobacillus delbrueckii* and *E. durans* were found to be positively associated with Sleep and Fatigue symptom severity in males, consistent with findings regarding Neurocognitive symptom severity. *L. delbrueckii* is also regarded as a probiotic, included in a probiotic preparation called VSL #3 [112]. While there do not appear to be any reports of *L. delbrueckii* being associated with psychological symptoms, the species has been linked to a case of pyelonephritis (kidney infection) and bacteremia [113], and urinary tract infections [114]. In females, *L. acidophilus*, also included in probiotic preparation VSL #3, was found to be positively associated with Sleep and Fatigue symptoms. These findings again draw attention to the need for further research into GM labelled as probiotics. Individuals may look to probiotics as an easy and safe supplement to improve their health, however these findings add weight to a growing body of evidence which suggest that, taken without consideration, probiotic supplements may worsen certain symptoms.

*Streptococcus* species were found to be negatively associated with Sleep and Fatigue symptom severity in both males (*S. mutans*) and females (*S. sanguinis*). *S. mutans* is typically regarded as a causative agent of tooth decay [115], and has also been associated with inflammatory bowel disorders [116]. *S. mutans* is able to metabolise a wide range of carbohydrates [117] which may be the mechanism through which it influences symptom expression. *S. sanguinis* is considered a commensal of the oral microbiota, but has been associated with endocarditis (infection of the valves or endocardial lining of the heart [118]) and potentially with cases of colonic carcinoma [119]. It is unclear how these species may be negatively associated with symptoms of Sleep and Fatigue in the current sample. Given the relatively small effect sizes, this may be due to statistical artifact. However, given that *S. mutans* has been demonstrated to be able to metabolise carbohydrates, further research is warranted.

As was the case with Neurocognitive symptoms, *B. bifidum* was negatively associated with Sleep and Fatigue symptom severity in males. Conversely, unidentified *Bifidobacterium* species were found to be positively associated with symptom severity in females. This is again suggestive that species belonging to the *Bifidobacterium* genus act in a sex dependent manner [32, 80]. *E. detmatitidis* was also again found to be negatively associated with Sleep and Fatigue symptom severity in males only.

### The clinical relevance of the microgenderome

The results of the current study in no way propose a causal link between GM and psychological symptom severity. However, consistent with previous research, the results provide further evidence for the microgenderome [31–33]. The pattern of relationships between GM and all four psychological symptom domains clearly varied by sex. This was despite all but one species which was related to symptom expression (*R. gnavus*) having a similar abundance in males and females. This finding was consistent with Wallis et al. [31] who also demonstrated sex-dependent relationships between GM and symptom expression despite similar GM composition. Taken together, these findings suggest that the role of sex hormones goes beyond organisational effects of GM, but also influences the action of specific species. Vemuri et al. [36] suggest that insight into the bidirectional interactions between sex hormones, GM, and immunity may provide valuable understanding of the sex discrepancies in susceptibility to psychological disorders.

Further to this point, it seems inappropriate to provide males and females with the same treatment and expect it to be equally efficacious. For example, sex differences have been noted in the efficacy of psychopharmacological treatment [120, 121]. Given that GM have been demonstrated to alter the bioavailability, bioactivity, or toxicity of drugs [122, 123] it is plausible that they may do so in a sex dependent manner. A study using a murine model found that *Ruminococcus flavefaciens* altered the effectiveness of antidepressant duloxetine. *Ruminoccocus* has been found to be more abundant in females [33, 67], while *R. gnavus* was found to be more abundant in males in the current study. Although inconsistent, these findings suggest that *Ruminoccocus* could be a genus particularly vulnerable to the effect of sex hormones. These findings suggest a need for further research into the potential interactions between psychopharmacological treatments and GM in males and females. While it is not proposed that GM modulation take the place of traditional psychopharmacological treatments, the microgenderome offers valuable insight into potentially enhancing treatment outcomes through the understanding of potential sex differences in GM-drug-host relationships. Before such auxiliary treatments are introduced, however, further research is needed.

### Limitations and future directions

The current study was limited by large amounts of missing data which precluded the ability to perform more sophisticated statistical analyses which could have provided a better picture of the intricate relationships between various species and symptoms. While the use of pairwise analysis was deemed necessary, this method of dealing with missing data can introduce bias in cases where data are not missing completely at random. As such, the focus of this study was not intended to be on any specific species, or suggesting that any specific species be used in the treatment of psychological disorders. Instead, the focus was more so on the pattern of relationships, and further exploring the concept of the microgenderome. Missing data also meant that the sample size available for certain correlations was small, meaning that statistical power was low. This could have undermined the true extent of the associations between GM and symptom severity. Missing data is a common issue in microbiota research and is complicated by the fact that it is unlike typical missing data. Referred to as “structural zeros”, missingness in microbiological data is due to an individual’s underlying biology [43]. This issue is further exacerbated by the fact that how this missingness, or structural zeros, is dealt with is not described in BGMA research studies. In a field that already has a number of different methodological procedures which may contribute to the discrepancies in previous research findings, the lack of clarity around how missingness is treated adds to potential methodological biases. It is imperative that a standardised method of dealing with missingness is developed and employed in BGMA research going forward.

A further limitation relates to the range of factors that were not controlled for, including but not limited to diet, exercise, medication, probiotic use, and underlying clinical diagnoses as this information was not available in the retrospectively collected dataset. As such, it was unclear whether any of these factors mediated the relationships found between GM and symptom severity. Future studies must make a concerted effort to control for as many extraneous variables that have been demonstrated to influence GM composition. Ideally, this should become the norm in BGMA research, as without controlling for factors that are known to influence GM, our understanding of GM-symptom associations will be incomplete. While large scale population studies remain useful in elucidating relationships between GM and symptom expression, smaller scale, well phenotyped and controlled longitudinal studies may facilitate greater control of numerous extraneous factors.

More generally, in addition to not controlling for, or considering, the multitude of factors that may influence GM composition, studies investigating the association between GM and psychological symptoms suffer from disparate methods of both microbial (culture-based methods, antibody-based assays, ribosomal RNA (16S rDNA) and shotgun metagenomic sequencing) and symptom data collection. Culture-based methods such as the one used in the current study counts only viable (live) cells, whereas sequencing techniques based on DNA cannot distinguish between live and dead cells [124]. An important question that has received little attention is whether dead cells impact on host health. The majority of research on this particular topic is in regards to the physiological functions of live probiotic species compared to the same heat killed species, but does demonstrate that non-viable (dead) bacteria may still influence host health [125–128]. These disparities in research methods may contribute to the inconsistencies found within the literature, therefore making it difficult to directly compare the findings across studies. While it may not always be practically feasible, it has been suggested that an ideal approach would be using culture-based methods alongside sequencing techniques, where the advantages of each together could outweigh the disadvantages of a single technique alone [129–131].

There are also several sampling methods for gut microbiota including stool sampling (most common) and biopsy, among others, with Tang et al. [132] calling for more precise measures of GM that are able to be used in large scale research and in healthy controls. This will inevitably be enabled by technological advances, as evidenced by the development of smart capsules, which hold promise as non-invasive accurate measures of intestinal microbiota [132, 133]. Going forward, efforts must be made for more standardised testing procedures. There is a need for more clinical trials with strict controls on extraneous variables, investigating particular psychological conditions. Given the immense interindividual differences that exist within the GM, longitudinal and repeated measures studies may also provide valuable insight into how shifts in GM relate to changes in symptom expression within the same individual. It is only once these pathways are established that GM modulation can be seriously considered in the diagnostic and treatment process.

In addition to investigating the bacterial component of the GM, future research must also consider the influence of other members of the GM such as viruses, fungi, protozoa, and helminths. While less numerous, these microbes may still have important effects on their human host. Ganci et al. [41] have taken an initial step in broadening the scope of BGMA research by being the first to investigate the effect of two common protozoan members of the GM (*Blastocystis* sp. and *Dientamoeba fragilis*) on psychological symptom severity. While the findings showed that these protozoa did not effect psychological symptom expression, further studies are called for before conclusions can be drawn. Following the current study, the next logical step for further research would be to consider the interactions between diverse members of the GM. This is important to consider as changes in the delicate balance of the gut ecosystem can change the fitness and pathogenic potential of otherwise commensal organisms. To understand the precise mechanisms of GM, research must consider the interplay between all members of the gut ecosystem.

## Conclusion

The results of the current study provide further evidence of associations between GM and psychological symptom expression in a human sample. Sex divergent patterns of these associations also provides additional support for the concept of the microgenderome. The results serve to suggest that a number of specific microbial species may hold promise in either providing auxiliary diagnostic information as potential biomarkers of health status as well as potential auxiliary treatment options. The findings of the current study also demonstrate the need for caution with regard to the indiscriminate use of probiotic supplements. Several species typically found in popular over the counter probiotic formulations were found to be positively associated with symptom severity. In some cases, the use of probiotics may do more harm than good. At this stage, it is not being suggested that probiotic use should be subject to the same restrictions as some medications, however there is a pressing need for further research into species currently considered probiotic.

The intersection of microbiology and psychology had remained largely distinct until the last decade or so [134]. This paper has demonstrated that the field of psychology can benefit from both the incorporation of GM research into the practice of clinical formulation, though also may be uniquely placed to inform microbiology research regarding the interactions between clinical symptoms and what this may mean for the GM. It is not the contention of this paper to suggest that psychologists should become well-versed in microbiology, nor is it suggested that all clients be referred to have their GM screened. To make the concept to the BGMA more accessible to practicing psychologists, Ganci et al. [135] related GM to each component of the Four P model of case formulation. In conducting clinical interviews, psychologists should consider asking questions around possible factors that may influence GM composition (such as a person’s diet or recent changes in diet), as these may be related to a client’s presenting problem, and to keep in mind that this may occur in a sex specific manner. It is particularly important to consider the possible role of GM when social and emotional precipitating or perpetuating factors are not present. In such cases, a multidisciplinary approach to treatment including a microbiologist may be advantageous. Inclusion of a complementary treatment targeting the GM would be in line with the growing push for personalised medicine, with the aim of tailoring an individual client’s treatment using a multidisciplinary approach which allows for a more holistic consideration of health and wellbeing. Before that is possible however, further research working towards understanding the precise mechanisms through which GM act on psychological symptom expression is needed.

## Supplementary Information

**Additional file 1.**

## Data Availability

The data that support the findings of this study are available from Bioscreen but restrictions apply to the availability of these data, which were used under licence for the current study, and so are not publicly available. Data are however available from the authors upon reasonable request and with permission of Bioscreen.
