# Supplementary material for: Associations between self-reported psychological symptom severity and gut microbiota: further support for the microgenderome
Source: BMC Psychiatry. 2022 Apr 30;22:307. doi: 10.1186/s12888-022-03947-7 (PMC9059404; doi:10.1186/s12888-022-03947-7)
Supplement: Supplementary file 1 — Additional file 1. [file 12888_2022_3947_MOESM1_ESM.docx]

**Supplemental Material 1**

Presented in Table S1 are the Kendall’s Tau-b (τ**_b_**) values and the associated *r*, *r*^2^, *n*, Fisher’s *z* transformation (*z_r_*) and *p* values for all significant associations between gut microbiota species and symptom severity.

**Table S1.**

*Conversion of Tau values to r values.*

| Microorganism | Tau | *r* | *Z*_r_ | *n* | *Z*obs | *r* square | *p* |
| --- | --- | --- | --- | --- | --- | --- | --- |
| Depressive symptoms (males) |  |  |  |  |  |  |  |
| *C. ramosum* | 0.184 | 0.285 | 0.586 | 65 |  | 0.081 | 0.036 |
| *L. lactis* | -0.446 | -0.645 | -1.532 | 13 |  | 0.416 | 0.034 |
| *S. mutans* | -0.097 | -0.152 | -0.306 | 220 |  | 0.023 | 0.036 |
| *B. vulgatus* | -0.057 | -0.089 | -0.179 | 700 |  | 0.008 | 0.029 |
| *B. bifidum* | -0.207 | -0.319 | -0.662 | 90 |  | 0.102 | 0.005 |
| *C. minutissimum* | -0.299 | -0.453 | -0.976 | 30 |  | 0.205 | 0.025 |
| Depressive symptoms (females) |  |  |  |  |  |  |  |
| *C. innocum* | -0.103 | -0.161 | -0.325 | 259 |  | 0.026 | 0.016 |
| *C. citroniae* | 0.439 | 0.636 | 1.504 | 19 |  | 0.405 | 0.009 |
| *C. hathewayi* | -0.157 | -0.244 | -0.498 | 82 |  | 0.060 | 0.041 |
| *E. avium* | 0.136 | 0.212 | 0.431 | 102 |  | 0.045 | 0.047 |
| *E. limosum* | 0.095 | 0.149 | 0.300 | 207 |  | 0.022 | 0.046 |
| *L. ruminis* | -0.246 | -0.377 | -0.793 | 38 |  | 0.142 | 0.035 |
| *S. dysgalactiae* | 0.259 | 0.396 | 0.837 | 56 |  | 0.157 | 0.006 |
| *B. massiliensis* | -0.11 | -0.172 | -0.347 | 237 |  | 0.030 | 0.014 |
| *C. aurimucosum* | 0.12 | 0.187 | 0.379 | 168 |  | 0.035 | 0.024 |
| *R. dentocariosa* | 0.12 | 0.187 | 0.379 | 140 |  | 0.035 | 0.04 |
| *A. oris* | 0.24 | 0.368 | 0.773 | 48 |  | 0.136 | 0.019 |
| Neurocognitive symptoms (males) |  |  |  |  |  |  |  |
| *C. tertium* | 0.205 | 0.331 | 0.688 | 62 |  | 0.110 | 0.021 |
| *E. durans* | 0.448 | 0.680 | 1.658 | 17 |  | 0.462 | 0.013 |
| *L. delbrueckii* | 0.357 | 0.550 | 1.237 | 25 |  | 0.303 | 0.014 |
| *L. lactis* | -0.543 | -0.852 | -2.527 | 13 |  | 0.726 | 0.012 |
| *A. shahii* | -0.17 | -0.265 | -0.543 | 119 |  | 0.070 | 0.007 |
| *B. bifidum* | -0.172 | -0.264 | -0.541 | 88 |  | 0.070 | 0.02 |
| *R. mucilaginosa* | 0.393 | 0.562 | 1.272 | 18 |  | 0.316 | 0.025 |
| Neurocognitive symptoms (females) |  |  |  |  |  |  |  |
| *E. durans* | -0.195 | -0.308 | -0.637 | 85 |  | 0.095 | 0.009 |
| *E. coli* | -0.027 | -0.042 | -0.084 | 3006 |  | 0.002 | 0.028 |
| *K. pnumoniae* | -0.085 | -0.125 | -0.251 | 395 |  | 0.016 | 0.013 |
| *C. parapsilosis* | 0.106 | 0.167 | 0.337 | 339 |  | 0.028 | 0.005 |
| *C. glabatra* | 0.22 | 0.339 | 0.705 | 50 |  | 0.115 | 0.027 |
| **Sex comaprison of E. durans** |  |  |  |  | 7.936 |  |  |
| Stress and Anxiety (males) |  |  |  |  |  |  |  |
| *L. plantarum* | 0.164 | 0.255 | 0.521 | 74 |  | 0.065 | 0.045 |
| *L. vaginalis* | -0.385 | -0.569 | -1.291 | 20 |  | 0.323 | 0.021 |
| *L. lactis* | -0.439 | -0.636 | -1.504 | 13 |  | 0.405 | 0.045 |
| *S. gallolyticus* | 0.487 | 0.693 | 1.706 | 19 |  | 0.480 | 0.006 |
| *A. shahii* | -0.15 | -0.233 | -0.476 | 119 |  | 0.054 | 0.019 |
| *E. dermatitidis* | -0.506 | -0.714 | -1.790 | 11 |  | 0.509 | 0.037 |
| Stress and Anxiety (females) |  |  |  |  |  |  |  |
| *C. innocum* | -0.09 | -0.141 | -0.284 | 252 |  | 0.020 | 0.038 |
| *E. durans* | -0.195 | -0.302 | -0.622 | 83 |  | 0.091 | 0.011 |
| *E. faecium* | -0.049 | -0.077 | -0.154 | 785 |  | 0.006 | 0.044 |
| *L. paracasei* | 0.069 | 0.108 | 0.217 | 392 |  | 0.012 | 0.046 |
| *L. lactis* | -0.275 | -0.419 | -0.892 | 28 |  | 0.175 | 0.043 |
| *R. gnavus* | -0.158 | -0.246 | -0.502 | 110 |  | 0.060 | 0.017 |
| *S. dysgalactiae* | 0.212 | 0.327 | 0.679 | 58 |  | 0.107 | 0.02 |
| **Sex comparison of L. lactis** |  |  |  |  | -1.634 |  |  |
| Sleep and Fatigue (males) |  |  |  |  |  |  |  |
| *L. delbrueckii* | 0.338 | 0.506 | 1.116 | 26 |  | 0.256 | 0.018 |
| *E. avium* | -0.246 | -0.377 | -0.793 | 33 |  | 0.142 | 0.048 |
| *E. durans* | 0.378 | 0.559 | 1.264 | 18 |  | 0.313 | 0.03 |
| *S. mutans* | -0.102 | -0.160 | -0.322 | 224 |  | 0.025 | 0.026 |
| *B. massiliensis* | 0.135 | 0.210 | 0.427 | 91 |  | 0.044 | 0.035 |
| *B. bifidum* | -0.249 | -0.381 | -0.803 | 90 |  | 0.145 | 0.001 |
| *E. dermatitidis* | -0.629 | -0.835 | -2.408 | 10 |  | 0.697 | 0.012 |
| Sleep and Fatigue (females) |  |  |  |  |  |  |  |
| *C. hathewayi* | -0.165 | -0.256 | -0.524 | 82 |  | 0.066 | 0.032 |
| *L. acidophilus* | 0.068 | 0.107 | 0.214 | 472 |  | 0.011 | 0.03 |
| *S. sanguinis* | -0.183 | -0.284 | -0.583 | 93 |  | 0.080 | 0.011 |
| *Bifidobacterium sp.* | 0.18 | 0.279 | 0.573 | 64 |  | 0.078 | 0.039 |
| *P. acnes* | 0.26 | 0.397 | 0.841 | 33 |  | 0.158 | 0.037 |
